# *trans*-1,2,3-Tris(4-methoxy­benzo­yl)cyclo­propane

**DOI:** 10.1107/S1600536809005145

**Published:** 2009-02-25

**Authors:** Jingjing Sun, Nengfang She

**Affiliations:** a Key Laboratory of Pesticides and Chemical Biology of the Ministry of Education, College of Chemistry, Central China Normal University, Wuhan 430079, People’s Republic of China

## Abstract

In the title compound, C_27_H_24_O_6_, the packing of the mol­ecules is mainly governed by C—H⋯O inter­actions.

## Related literature

For related structures, see: Saba (1990 ▶). For background to the chemistry of cyclo­propanes as a versatile tool in organic synthesis, see: Wong (1989 ▶).
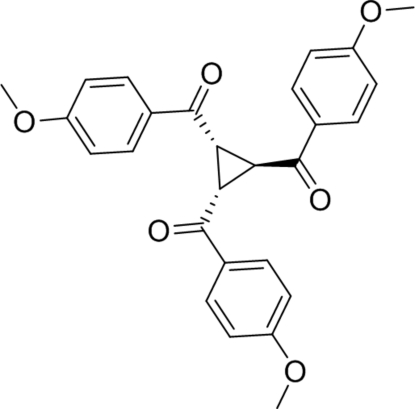


## Experimental

### 

#### Crystal data

C_27_H_24_O_6_
*M* *_r_* = 444.46
Triclinic, 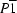

*a* = 10.1897 (6) Å
*b* = 10.626 (6) Å
*c* = 10.6931 (6) Å
α = 90.736 (1)°
β = 103.194 (1)°
γ = 92.432 (1)°
*V* = 1125.9 (6) Å^3^
*Z* = 2
Mo *K*α radiation
μ = 0.09 mm^−1^
*T* = 298 K
0.30 × 0.20 × 0.10 mm

#### Data collection

Bruker SMART CCD area-detector diffractometer
Absorption correction: none
9852 measured reflections
4349 independent reflections
2862 reflections with *I* > 2σ(*I*)
*R*_int_ = 0.031

#### Refinement

*R*[*F* ^2^ > 2σ(*F* ^2^)] = 0.057
*wR*(*F* ^2^) = 0.155
*S* = 1.06
4349 reflections
301 parameters
H-atom parameters constrained
Δρ_max_ = 0.19 e Å^−3^
Δρ_min_ = −0.17 e Å^−3^

### 

Data collection: *SMART* (Bruker, 2001 ▶); cell refinement: *SAINT* (Bruker, 2001 ▶); data reduction: *SAINT*; program(s) used to solve structure: *SHELXS97* (Sheldrick, 2008 ▶); program(s) used to refine structure: *SHELXL97* (Sheldrick, 2008 ▶); molecular graphics: *SHELXTL* (Sheldrick, 2008 ▶); software used to prepare material for publication: *SHELXTL*.

## Supplementary Material

Crystal structure: contains datablocks global, I. DOI: 10.1107/S1600536809005145/rk2123sup1.cif

Structure factors: contains datablocks I. DOI: 10.1107/S1600536809005145/rk2123Isup2.hkl

Additional supplementary materials:  crystallographic information; 3D view; checkCIF report

## Figures and Tables

**Table 1:** Hydrogen-bond geometry (Å, °)

*D*—H⋯*A*	*D*—H	H⋯*A*	*D*⋯*A*	*D*—H⋯*A*
C26—H26⋯O2^i^	0.93	2.54	3.467 (3)	175
C11—H11⋯O6^ii^	0.98	2.51	3.270 (3)	134
C7—H7⋯O5^iii^	0.93	2.52	3.171 (3)	128
C9—H9⋯O3^iii^	0.98	2.55	3.497 (3)	164
C27—H27*B*⋯O4^iv^	0.96	2.58	3.136 (3)	117

Symmetry codes: (i) 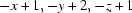; (ii) 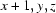; (iii) 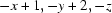; (iv) 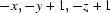.

**Fig. 1.**
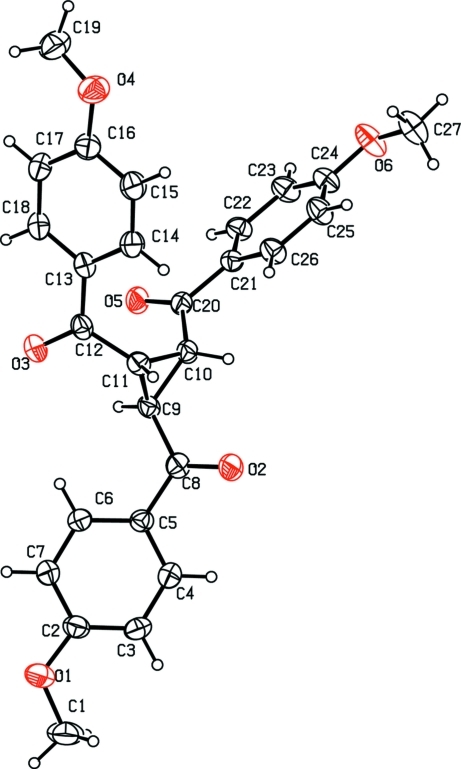
The molecular structure of the title compound with the atom–numbering scheme. Displacement ellipsoids are drawn at the 30% probability level. H atoms are presented as a small spheres of arbitrary radius.

## supplementary crystallographic information

### Comment

The unusual bonding of cyclopropanes and the strain release associated with cleavage of cyclopropanes offer the possibility of recognizing that structural unit when it is a part of a larger molecule. We report here the molecular structure of the title cyclopropane derivative (Fig. 1), which is an important intermediate in organic synthesis (Saba, 1990). Since numerous methodologies have been developed for the construction of three–membered carbocycles, the chemistry of cyclopropanes has emerged as a versatile tool in organic synthesis (Wong, 1989).

The crystal packing is stabilized by intermolecular C—H···O interaction.

### Experimental

The title compound was synthesized according to the procedure reported (Saba, 1990). Crystals appropriate for X–ray data collection were obtained by slow evaporation of the dichloromethane solution at 293 K.

### Refinement

All H atoms were positioned in geometrically idealized positions and constrained to ride on their parent atoms, with C—H distances in the range 0.93–0.98 Å with *U*_iso_(H) = 1.2*U*_eq_(C) or *U*_iso_(H) = 1.5*U*_eq_(C).

### Crystal data

**Table:**

C_27_H_24_O_6_	*Z* = 2
*M**_r_* = 444.46	*F*(000) = 468
Triclinic, *P*1	*D*_x_ = 1.311 Mg m^−^^3^
Hall symbol: -P 1	Mo *K*α radiation, λ = 0.71073 Å
*a* = 10.1897 (6) Å	Cell parameters from 1774 reflections
*b* = 10.626 (6) Å	θ = 2.7–21.3°
*c* = 10.6931 (6) Å	µ = 0.09 mm^−^^1^
α = 90.736 (1)°	*T* = 298 K
β = 103.194 (1)°	Block, colourless
γ = 92.432 (1)°	0.30 × 0.20 × 0.10 mm
*V* = 1125.9 (6) Å^3^	

### Data collection

**Table:**

Bruker SMART CCD area-detector diffractometer	2862 reflections with *I* > 2σ(*I*)
Radiation source: Fine–focus sealed tube	*R*_int_ = 0.031
Graphite	θ_max_ = 26.0°, θ_min_ = 1.9°
φ and ω scans	*h* = −12→12
9852 measured reflections	*k* = −10→13
4349 independent reflections	*l* = −13→13

### Refinement

**Table:**

Refinement on *F*^2^	Primary atom site location: Direct
Least-squares matrix: Full	Secondary atom site location: Difmap
*R*[*F*^2^ > 2σ(*F*^2^)] = 0.057	Hydrogen site location: Geom
*wR*(*F*^2^) = 0.155	H-atom parameters constrained
*S* = 1.06	*w* = 1/[σ^2^(*F*_o_^2^) + (0.0697*P*)^2^ + 0.0779*P*] where *P* = (*F*_o_^2^ + 2*F*_c_^2^)/3
4349 reflections	(Δ/σ)_max_ = 0.001
301 parameters	Δρ_max_ = 0.19 e Å^−^^3^
0 restraints	Δρ_min_ = −0.17 e Å^−^^3^

### Special details

**Table:**

Geometry. All s.u.'s (except the s.u. in the dihedral angle between two l.s. planes) are estimated using the full covariance matrix. The cell s.u.'s are taken into account individually in the estimation of s.u.'s in distances, angles and torsion angles; correlations between s.u.'s in cell parameters are only used when they are defined by crystal symmetry. An approximate (isotropic) treatment of cell s.u.'s is used for estimating s.u.'s involving l.s. planes.
Refinement. Refinement of *F*^2^ against ALL reflections. The weighted *R*–factor *wR* and goodness of fit *S* are based on *F*^2^, conventional *R*–factors *R* are based on *F*, with *F* set to zero for negative *F*^2^. The threshold expression of *F*^2^ > σ(*F*^2^) is used only for calculating *R*–factors(gt) *etc*. and is not relevant to the choice of reflections for refinement. *R*–factors based on *F*^2^ are statistically about twice as large as those based on *F* and *R*–factors based on ALL data will be even larger.

### Fractional atomic coordinates and isotropic or equivalent isotropic displacement parameters (Å^2^)

**Table:**

	*x*	*y*	*z*	*U*_iso_*/*U*_eq_	
C1	1.0074 (3)	1.5583 (3)	0.2073 (3)	0.0938 (10)	
H1A	0.9340	1.6141	0.1903	0.141*	
H1B	1.0773	1.5892	0.1672	0.141*	
H1C	1.0425	1.5543	0.2983	0.141*	
C2	0.8628 (2)	1.3733 (2)	0.2028 (2)	0.0559 (6)	
C3	0.8010 (3)	1.4209 (2)	0.2944 (2)	0.0632 (7)	
H3	0.8256	1.5011	0.3302	0.076*	
C4	0.7018 (3)	1.3478 (2)	0.3320 (2)	0.0598 (7)	
H4	0.6627	1.3784	0.3961	0.072*	
C5	0.6594 (2)	1.2303 (2)	0.27708 (19)	0.0452 (6)	
C6	0.7213 (2)	1.1851 (2)	0.1834 (2)	0.0480 (6)	
H6	0.6935	1.1070	0.1440	0.058*	
C7	0.8233 (2)	1.2554 (2)	0.1489 (2)	0.0578 (7)	
H7	0.8662	1.2229	0.0884	0.069*	
C8	0.5550 (2)	1.1548 (2)	0.32226 (19)	0.0489 (6)	
C9	0.4782 (2)	1.0509 (2)	0.23775 (18)	0.0447 (6)	
H9	0.4560	1.0668	0.1454	0.054*	
C10	0.3734 (2)	0.9763 (2)	0.28795 (19)	0.0453 (6)	
H10	0.3694	0.9979	0.3763	0.054*	
C11	0.5019 (2)	0.9174 (2)	0.27438 (19)	0.0452 (6)	
H11	0.5681	0.9061	0.3552	0.054*	
C12	0.5012 (2)	0.8198 (2)	0.1703 (2)	0.0480 (6)	
C13	0.4473 (2)	0.6911 (2)	0.1861 (2)	0.0467 (6)	
C14	0.4281 (3)	0.6476 (3)	0.3027 (2)	0.0592 (7)	
H14	0.4515	0.6999	0.3756	0.071*	
C15	0.3747 (3)	0.5276 (3)	0.3114 (2)	0.0684 (8)	
H15	0.3643	0.4989	0.3905	0.082*	
C16	0.3365 (3)	0.4493 (2)	0.2036 (2)	0.0571 (6)	
C17	0.3553 (3)	0.4921 (3)	0.0870 (2)	0.0578 (7)	
H17	0.3294	0.4409	0.0135	0.069*	
C18	0.4123 (2)	0.6105 (2)	0.0802 (2)	0.0529 (6)	
H18	0.4278	0.6372	0.0021	0.064*	
C19	0.2147 (3)	0.2623 (3)	0.1091 (3)	0.0829 (9)	
H19A	0.1527	0.3131	0.0523	0.124*	
H19B	0.1663	0.1912	0.1343	0.124*	
H19C	0.2810	0.2334	0.0659	0.124*	
C20	0.2404 (2)	0.9402 (2)	0.20001 (19)	0.0437 (6)	
C21	0.1269 (2)	0.9056 (2)	0.25933 (19)	0.0425 (5)	
C22	−0.0052 (2)	0.9130 (2)	0.1882 (2)	0.0503 (6)	
H22	−0.0211	0.9394	0.1037	0.060*	
C23	−0.1126 (2)	0.8816 (2)	0.2419 (2)	0.0582 (7)	
H23	−0.2003	0.8875	0.1936	0.070*	
C24	−0.0906 (2)	0.8411 (2)	0.3674 (2)	0.0538 (6)	
C25	0.0391 (2)	0.8319 (3)	0.4392 (2)	0.0593 (7)	
H25	0.0545	0.8039	0.5231	0.071*	
C26	0.1462 (2)	0.8650 (2)	0.3847 (2)	0.0534 (6)	
H26	0.2337	0.8598	0.4337	0.064*	
C27	−0.1880 (3)	0.7676 (3)	0.5382 (3)	0.0860 (9)	
H27A	−0.1411	0.6906	0.5457	0.129*	
H27B	−0.2753	0.7524	0.5563	0.129*	
H27C	−0.1372	0.8295	0.5982	0.129*	
O2	0.53287 (17)	1.17456 (18)	0.42848 (14)	0.0672 (5)	
O1	0.96041 (19)	1.43598 (18)	0.15687 (19)	0.0816 (6)	
O3	0.54286 (18)	0.84862 (17)	0.07605 (15)	0.0635 (5)	
O4	0.2801 (2)	0.33557 (18)	0.22053 (17)	0.0786 (6)	
O5	0.22625 (15)	0.94253 (16)	0.08352 (13)	0.0579 (5)	
O6	−0.20364 (17)	0.8128 (2)	0.41048 (18)	0.0797 (6)	

### Atomic displacement parameters (Å^2^)

**Table:**

	*U*^11^	*U*^22^	*U*^33^	*U*^12^	*U*^13^	*U*^23^
C1	0.086 (2)	0.058 (2)	0.140 (3)	−0.0223 (17)	0.036 (2)	−0.0041 (19)
C2	0.0474 (14)	0.0519 (16)	0.0689 (15)	−0.0061 (12)	0.0160 (12)	0.0002 (12)
C3	0.0651 (16)	0.0477 (17)	0.0761 (17)	−0.0103 (13)	0.0178 (13)	−0.0154 (13)
C4	0.0622 (15)	0.0631 (18)	0.0557 (14)	−0.0040 (13)	0.0191 (12)	−0.0128 (12)
C5	0.0447 (12)	0.0474 (15)	0.0409 (11)	−0.0032 (11)	0.0057 (9)	0.0003 (10)
C6	0.0504 (13)	0.0439 (14)	0.0487 (12)	−0.0027 (11)	0.0103 (10)	−0.0032 (10)
C7	0.0532 (14)	0.0607 (18)	0.0636 (15)	−0.0030 (13)	0.0233 (12)	−0.0097 (12)
C8	0.0461 (13)	0.0625 (17)	0.0343 (11)	0.0013 (11)	0.0016 (9)	0.0022 (10)
C9	0.0431 (12)	0.0574 (16)	0.0320 (10)	−0.0077 (11)	0.0073 (9)	0.0032 (10)
C10	0.0432 (12)	0.0569 (15)	0.0354 (10)	−0.0034 (11)	0.0094 (9)	−0.0037 (10)
C11	0.0382 (12)	0.0564 (16)	0.0388 (11)	−0.0011 (10)	0.0047 (9)	0.0037 (10)
C12	0.0381 (12)	0.0635 (17)	0.0435 (12)	0.0040 (11)	0.0115 (10)	0.0020 (11)
C13	0.0441 (12)	0.0542 (16)	0.0445 (12)	0.0069 (11)	0.0149 (10)	0.0039 (10)
C14	0.0696 (17)	0.0643 (19)	0.0442 (13)	−0.0035 (14)	0.0154 (11)	0.0006 (11)
C15	0.091 (2)	0.071 (2)	0.0454 (13)	−0.0064 (16)	0.0213 (13)	0.0065 (13)
C16	0.0659 (16)	0.0509 (17)	0.0565 (14)	0.0060 (13)	0.0171 (12)	0.0070 (12)
C17	0.0695 (17)	0.0575 (18)	0.0483 (13)	0.0088 (13)	0.0168 (12)	−0.0048 (12)
C18	0.0595 (15)	0.0570 (17)	0.0471 (13)	0.0121 (13)	0.0205 (11)	0.0042 (11)
C19	0.099 (2)	0.061 (2)	0.086 (2)	−0.0007 (17)	0.0178 (17)	−0.0101 (15)
C20	0.0434 (12)	0.0473 (15)	0.0403 (12)	0.0001 (10)	0.0101 (9)	−0.0047 (10)
C21	0.0397 (12)	0.0444 (14)	0.0428 (11)	−0.0024 (10)	0.0089 (9)	−0.0008 (10)
C22	0.0469 (13)	0.0543 (16)	0.0471 (12)	−0.0035 (11)	0.0063 (10)	0.0066 (11)
C23	0.0384 (13)	0.0705 (18)	0.0630 (15)	0.0004 (12)	0.0056 (11)	0.0106 (13)
C24	0.0415 (13)	0.0589 (17)	0.0637 (15)	0.0017 (11)	0.0169 (11)	0.0124 (12)
C25	0.0526 (15)	0.0770 (19)	0.0490 (13)	0.0006 (13)	0.0128 (11)	0.0148 (12)
C26	0.0381 (12)	0.0714 (18)	0.0478 (13)	−0.0002 (12)	0.0042 (10)	0.0080 (12)
C27	0.0723 (19)	0.111 (3)	0.086 (2)	0.0035 (18)	0.0399 (16)	0.0242 (18)
O2	0.0693 (11)	0.0922 (14)	0.0383 (9)	−0.0202 (10)	0.0137 (8)	−0.0112 (8)
O1	0.0740 (13)	0.0648 (14)	0.1145 (15)	−0.0186 (10)	0.0440 (11)	−0.0110 (11)
O3	0.0715 (11)	0.0723 (13)	0.0538 (9)	−0.0054 (9)	0.0307 (8)	0.0021 (8)
O4	0.1083 (16)	0.0589 (13)	0.0671 (11)	−0.0104 (11)	0.0191 (10)	0.0065 (9)
O5	0.0540 (10)	0.0799 (13)	0.0384 (8)	−0.0060 (9)	0.0097 (7)	−0.0086 (8)
O6	0.0481 (10)	0.1137 (17)	0.0840 (13)	0.0054 (10)	0.0268 (9)	0.0337 (11)

### Geometric parameters (Å, °)

**Table:**

C1—O1	1.422 (3)	C14—C15	1.378 (3)
C1—H1A	0.9600	C14—H14	0.9300
C1—H1B	0.9600	C15—C16	1.383 (3)
C1—H1C	0.9600	C15—H15	0.9300
C2—O1	1.360 (3)	C16—O4	1.348 (3)
C2—C7	1.377 (3)	C16—C17	1.384 (3)
C2—C3	1.381 (3)	C17—C18	1.373 (3)
C3—C4	1.383 (3)	C17—H17	0.9300
C3—H3	0.9300	C18—H18	0.9300
C4—C5	1.381 (3)	C19—O4	1.428 (3)
C4—H4	0.9300	C19—H19A	0.9600
C5—C6	1.390 (3)	C19—H19B	0.9600
C5—C8	1.477 (3)	C19—H19C	0.9600
C6—C7	1.376 (3)	C20—O5	1.222 (2)
C6—H6	0.9300	C20—C21	1.477 (3)
C7—H7	0.9300	C21—C26	1.387 (3)
C8—O2	1.225 (2)	C21—C22	1.393 (3)
C8—C9	1.492 (3)	C22—C23	1.378 (3)
C9—C11	1.491 (3)	C22—H22	0.9300
C9—C10	1.503 (3)	C23—C24	1.387 (3)
C9—H9	0.9800	C23—H23	0.9300
C10—C20	1.494 (3)	C24—O6	1.358 (3)
C10—C11	1.510 (3)	C24—C25	1.376 (3)
C10—H10	0.9800	C25—C26	1.384 (3)
C11—C12	1.510 (3)	C25—H25	0.9300
C11—H11	0.9800	C26—H26	0.9300
C12—O3	1.218 (2)	C27—O6	1.430 (3)
C12—C13	1.478 (3)	C27—H27A	0.9600
C13—C18	1.382 (3)	C27—H27B	0.9600
C13—C14	1.387 (3)	C27—H27C	0.9600
			
O1—C1—H1A	109.5	C15—C14—C13	120.5 (2)
O1—C1—H1B	109.5	C15—C14—H14	119.8
H1A—C1—H1B	109.5	C13—C14—H14	119.8
O1—C1—H1C	109.5	C14—C15—C16	120.7 (2)
H1A—C1—H1C	109.5	C14—C15—H15	119.6
H1B—C1—H1C	109.5	C16—C15—H15	119.6
O1—C2—C7	115.4 (2)	O4—C16—C15	116.4 (2)
O1—C2—C3	124.8 (2)	O4—C16—C17	124.5 (2)
C7—C2—C3	119.8 (2)	C15—C16—C17	119.1 (2)
C2—C3—C4	119.1 (2)	C18—C17—C16	119.7 (2)
C2—C3—H3	120.5	C18—C17—H17	120.2
C4—C3—H3	120.5	C16—C17—H17	120.2
C5—C4—C3	121.8 (2)	C17—C18—C13	121.8 (2)
C5—C4—H4	119.1	C17—C18—H18	119.1
C3—C4—H4	119.1	C13—C18—H18	119.1
C4—C5—C6	118.1 (2)	O4—C19—H19A	109.5
C4—C5—C8	119.8 (2)	O4—C19—H19B	109.5
C6—C5—C8	122.0 (2)	H19A—C19—H19B	109.5
C7—C6—C5	120.3 (2)	O4—C19—H19C	109.5
C7—C6—H6	119.8	H19A—C19—H19C	109.5
C5—C6—H6	119.8	H19B—C19—H19C	109.5
C6—C7—C2	120.8 (2)	O5—C20—C21	121.68 (18)
C6—C7—H7	119.6	O5—C20—C10	120.80 (19)
C2—C7—H7	119.6	C21—C20—C10	117.50 (17)
O2—C8—C5	121.07 (19)	C26—C21—C22	117.9 (2)
O2—C8—C9	119.9 (2)	C26—C21—C20	122.47 (19)
C5—C8—C9	119.03 (18)	C22—C21—C20	119.67 (19)
C11—C9—C8	119.55 (18)	C23—C22—C21	120.6 (2)
C11—C9—C10	60.57 (15)	C23—C22—H22	119.7
C8—C9—C10	117.15 (17)	C21—C22—H22	119.7
C11—C9—H9	116.0	C22—C23—C24	120.4 (2)
C8—C9—H9	116.0	C22—C23—H23	119.8
C10—C9—H9	116.0	C24—C23—H23	119.8
C20—C10—C9	119.72 (17)	O6—C24—C25	124.6 (2)
C20—C10—C11	121.69 (18)	O6—C24—C23	115.4 (2)
C9—C10—C11	59.33 (14)	C25—C24—C23	120.0 (2)
C20—C10—H10	114.9	C24—C25—C26	119.1 (2)
C9—C10—H10	114.9	C24—C25—H25	120.5
C11—C10—H10	114.9	C26—C25—H25	120.5
C9—C11—C10	60.10 (15)	C25—C26—C21	122.0 (2)
C9—C11—C12	118.96 (18)	C25—C26—H26	119.0
C10—C11—C12	121.17 (17)	C21—C26—H26	119.0
C9—C11—H11	115.2	O6—C27—H27A	109.5
C10—C11—H11	115.2	O6—C27—H27B	109.5
C12—C11—H11	115.2	H27A—C27—H27B	109.5
O3—C12—C13	121.9 (2)	O6—C27—H27C	109.5
O3—C12—C11	119.9 (2)	H27A—C27—H27C	109.5
C13—C12—C11	118.13 (19)	H27B—C27—H27C	109.5
C18—C13—C14	118.1 (2)	C2—O1—C1	119.2 (2)
C18—C13—C12	119.0 (2)	C16—O4—C19	118.2 (2)
C14—C13—C12	122.9 (2)	C24—O6—C27	118.2 (2)
			
O1—C2—C3—C4	179.4 (2)	C18—C13—C14—C15	−0.5 (4)
C7—C2—C3—C4	1.2 (4)	C12—C13—C14—C15	178.6 (2)
C2—C3—C4—C5	−2.5 (4)	C13—C14—C15—C16	−1.4 (4)
C3—C4—C5—C6	1.4 (4)	C14—C15—C16—O4	−177.3 (2)
C3—C4—C5—C8	178.9 (2)	C14—C15—C16—C17	1.4 (4)
C4—C5—C6—C7	1.1 (3)	O4—C16—C17—C18	179.2 (2)
C8—C5—C6—C7	−176.4 (2)	C15—C16—C17—C18	0.6 (4)
C5—C6—C7—C2	−2.3 (4)	C16—C17—C18—C13	−2.5 (4)
O1—C2—C7—C6	−177.2 (2)	C14—C13—C18—C17	2.5 (4)
C3—C2—C7—C6	1.1 (4)	C12—C13—C18—C17	−176.6 (2)
C4—C5—C8—O2	−21.4 (3)	C9—C10—C20—O5	18.0 (3)
C6—C5—C8—O2	156.0 (2)	C11—C10—C20—O5	−52.3 (3)
C4—C5—C8—C9	160.2 (2)	C9—C10—C20—C21	−160.3 (2)
C6—C5—C8—C9	−22.4 (3)	C11—C10—C20—C21	129.4 (2)
O2—C8—C9—C11	−68.5 (3)	O5—C20—C21—C26	159.8 (2)
C5—C8—C9—C11	109.9 (2)	C10—C20—C21—C26	−21.9 (3)
O2—C8—C9—C10	1.3 (3)	O5—C20—C21—C22	−20.1 (3)
C5—C8—C9—C10	179.8 (2)	C10—C20—C21—C22	158.2 (2)
C11—C9—C10—C20	−111.4 (2)	C26—C21—C22—C23	0.4 (3)
C8—C9—C10—C20	138.3 (2)	C20—C21—C22—C23	−179.7 (2)
C8—C9—C10—C11	−110.3 (2)	C21—C22—C23—C24	−0.5 (4)
C8—C9—C11—C10	106.4 (2)	C22—C23—C24—O6	179.9 (2)
C8—C9—C11—C12	−142.2 (2)	C22—C23—C24—C25	−0.1 (4)
C10—C9—C11—C12	111.4 (2)	O6—C24—C25—C26	−179.3 (2)
C20—C10—C11—C9	108.1 (2)	C23—C24—C25—C26	0.7 (4)
C20—C10—C11—C12	0.4 (3)	C24—C25—C26—C21	−0.8 (4)
C9—C10—C11—C12	−107.8 (2)	C22—C21—C26—C25	0.3 (4)
C9—C11—C12—O3	33.7 (3)	C20—C21—C26—C25	−179.6 (2)
C10—C11—C12—O3	104.4 (3)	C7—C2—O1—C1	−179.9 (3)
C9—C11—C12—C13	−145.5 (2)	C3—C2—O1—C1	1.9 (4)
C10—C11—C12—C13	−74.8 (3)	C15—C16—O4—C19	167.5 (2)
O3—C12—C13—C18	−16.1 (3)	C17—C16—O4—C19	−11.1 (4)
C11—C12—C13—C18	163.0 (2)	C25—C24—O6—C27	−1.4 (4)
O3—C12—C13—C14	164.8 (2)	C23—C24—O6—C27	178.6 (3)
C11—C12—C13—C14	−16.0 (3)		

### Hydrogen-bond geometry (Å, °)

**Table:**

*D*—H···*A*	*D*—H	H···*A*	*D*···*A*	*D*—H···*A*
C26—H26···O2^i^	0.93	2.54	3.467 (3)	175
C11—H11···O6^ii^	0.98	2.51	3.270 (3)	134
C7—H7···O5^iii^	0.93	2.52	3.171 (3)	128
C9—H9···O3^iii^	0.98	2.55	3.497 (3)	164
C27—H27B···O4^iv^	0.96	2.58	3.136 (3)	117

Symmetry codes: (i) −*x*+1, −*y*+2, −*z*+1; (ii) *x*+1, *y*, *z*; (iii) −*x*+1, −*y*+2, −*z*; (iv) −*x*, −*y*+1, −*z*+1.
